# Responsiveness of a simple tool for assessing change in behavioral intention after continuing professional development activities

**DOI:** 10.1371/journal.pone.0176678

**Published:** 2017-05-01

**Authors:** France Légaré, Adriana Freitas, Stéphane Turcotte, Francine Borduas, André Jacques, Francesca Luconi, Gaston Godin, Andrée Boucher, Joan Sargeant, Michel Labrecque

**Affiliations:** 1 CHU de Québec Research Centre, Quebec City, Quebec, Canada; 2 Department of Family Medicine and Emergency Medicine, Université Laval, Quebec City, Canada; 3 Office of the Vice-Dean of Education and Continuing Professional Development, Faculty of Medicine, Université Laval, Quebec, Quebec, Canada; 4 Advisor in Continuing Professional Development, Saint-Adolphe-d’Howard, Quebec, Canada; 5 Continuing Health Professional Education Office, Faculty of Medicine, McGill University, Montreal, Quebec, Canada; 6 Faculty of Nursing, Université Laval, Quebec City, Quebec, Canada; 7 Centre de pédagogie appliquée aux sciences de la santé, Faculty of Medicine, Université de Montréal, Quebec, Canada; 8 Division of Medical Education, Faculty of Medicine, Dalhousie University, Nova Scotia, Canada; Royal College of Physicians and Surgeons of Canada, CANADA

## Abstract

**Background:**

Continuing professional development (CPD) activities are one way that new knowledge can be translated into changes in practice. However, few tools are available for evaluating the extent to which these activities change health professionals’ behavior. We developed a questionnaire called CPD-Reaction for assessing the impact of CPD activities on health professionals’ clinical behavioral intentions. We evaluated its responsiveness to change in behavioral intention and verified its acceptability among stakeholders.

**Methods and findings:**

We enrolled 376 health professionals who completed CPD-Reaction before and immediately after attending a CPD activity. We contacted them three months later and asked them to self-report on any behavior change. We compared the mean rankings on each CPD-Reaction construct before and immediately after CPD activities. To estimate its predictive validity, we compared the median behavioral intention score (post-activity) of health professionals reporting a behavior change three months later with the median behavioral intention score of physicians who reported no change. We explored stakeholders’ views on CPD-Reaction in semi-structured interviews. Participants were mostly family physicians (62.2%), with an average of 19 years of clinical practice. Post-activity, we observed an increase in intention-related scores for all constructs (*P* < 0.001) with the most appreciable for the construct *beliefs about capabilities*. A total of 313 participants agreed to be contacted at follow up, and of these only 69 (22%) reported back. Of these, 43 (62%) self-reported a behavior change. We observed no statistically significant difference in intention between health professionals who later reported a behavior change and those who reported no change (*P* = 0.30). Overall, CPD stakeholders found the CPD-Reaction questionnaire of interest and suggested potential solutions to perceived barriers to its implementation.

**Conclusion:**

The CPD-Reaction questionnaire seems responsive to change in behavioral intention. Although CPD stakeholders found it interesting, future implementation will require addressing barriers they identified.

## Introduction

Participation in continuing professional development (CPD) training programs for health professionals forms a central component of quality improvement efforts [1–4]. A 2016 scoping review of national programs to validate physician competence and fitness for practice showed an emerging concern that, owing to the rapid increase in evidence relevant to practice, continuing education and assessment should be a requirement for today’s physicians [5]. In a survey of 10 European countries, all of them viewed CPD as an important mechanism for demonstrating that doctors continue to meet key standards [6]. CPD training programs are now expected to transmit new knowledge in ways that enable health professionals not only to gain new knowledge but to change their behavior in the clinic so that patient care is optimal and patient outcomes improve [7,8].

However, a lack of theory hampers these efforts. Studies included in the 2016 scoping review of national programs frequently cited goals and aims, for example, but none described any theoretical framework used to guide setting up the program or reported on evidence used to inform program design [5]. Indeed, there are few relevant conceptual models for understanding behavior change in health professionals and thus little is known about how to design effective interventions for CPD programs [9–11]. Some conceptual CPD frameworks have been developed in reaction to evidence that a) CPD activities should be based on assessed need, b) passive approaches do not generally change physician behavior, and c) interactive activities (with practice and feedback) are more effective. Moore et al., for example, developed their “expanded outcomes framework” for planning and assessing continuous learning [12]. Taking six assessment criteria from an earlier model (participation, satisfaction, learning, performance, patient health and community health) they expanded its learning/performance components into Miller’s 4-level formula: “knows”, “knows how”, “shows how”, and “does” [13]. Few conceptual models for CPD have integrated the knowledge base of socio-cognitive behavior change theories, however [14–16], and there are few valid and reliable methods for identifying the factors influencing health professionals’ behavior in this context [17]. Together, these gaps in knowledge may partially explain why so few CPD activities have proven effective for promoting behavior change among health professionals [18,19].

In 2009, CPD decision makers and knowledge translation researchers in Canada met to begin the process of developing a short, theory-based questionnaire that could be used as a tool for routinely assessing the impact of CPD activities on clinical practice [19,20]. The CPD-Reaction questionnaire shows adequate validity and reliability, with Cronbach’s coefficients for the constructs varying from 0.77 to 0.85 [19]. It is a 12-item instrument based on an integrated model combining a number of social cognitive theories for explaining health professionals’ clinical behavior through the proxy of intention (e.g. the Theory of Planned Behavior and Triandis’ theory) [11]. Briefly, this integrated model proposes that three categories of variables predict the behavior of health professionals: 1) their intention to adopt a particular behavior or not; 2) their beliefs about their capabilities; and 3) their past behavior and habits. The model also suggests that the first category, behavioral intention, is influenced by several factors as described in the original account of the model[11].

This study describes the validity of the CPD-Reaction questionnaire for assessing the impact of CPD activities on health professionals’ clinical behavioral intentions [19,20]. Specifically, we sought to evaluate the responsiveness of the CPD-Reaction questionnaire (i.e., its ability to measure changes in behavioral intention after a CPD activity) and its predictive potential for subsequent behavior change. We also explored its acceptability among CPD providers to develop an implementation plan for its use in CPD activities.

## Methods

### Ethics statement

The Research Ethics Committee of the Centre Hospitalier Universitaire de Québec (CHU de Québec) approved this project on 30 June, 2010 (project # S10-06-033). Participants gave written informed consent to participate in the study. The Ethics Committee approved the consent procedure.

### Study design

We conducted a prospective mixed-methods study. First, we performed a before-and-after study with health professionals attending accredited CPD activities to evaluate the CPD-Reaction questionnaire’s responsiveness to change in behavioral intention and its predictive validity (subsequent self-reported behavior change). We also conducted a qualitative study with CPD decision-makers and providers to verify its acceptability. A detailed protocol of the entire research project is published elsewhere [20].

### Quantitative study

#### Participants and recruitment strategy

We recruited participants who attended eligible CPD activities offered by the CPD providers and collaborators in this project [19]. Eligibility criteria for CPD activities included the following: (a) be accredited by one of Quebec’s CPD providers; (b) be group-based, with live activities; (c) be focused on one main behavior change as stated in the learning objectives established for that activity, (d) take place in any setting; (e) use any materials and teaching methods or combination thereof; and (f) are one-time interventions. Activities offered as part of a larger program, such as a medical conference held over several days, were also eligible. Eligibility criteria for participating health professionals were: (a) attending an eligible live CPD activity; (b) is still in clinical practice six-months after the indexed CPD activity; (c) agrees to be contacted for a phone interview three months after the indexed CPD activity; (d) speaks French or English; and (e) has not participated in this study before.

#### Data collection

Research assistants (RAs) attended those CPD activities that were eligible from among all the activities available [19]. As soon as attendees arrived at the CPD activity, RAs invited them to participate in the study. Those who agreed to participate were asked to complete a consent form and the CPD-Reaction questionnaire, a self-administered paper-based questionnaire adapted to match each eligible CPD activity. The generic CPD-Reaction questionnaire was adapted by replacing the word “behavior” in each item with an observable or measurable learning objective from the CPD activity in question (e.g., “handling an automated external defibrillator”, see S1 Table). Participants completed the CPD-Reaction questionnaire just before and immediately after the CPD activity. Data was collected anonymously. Participants were then contacted three months later by email to collect information about their self-reported clinical behavior. At that moment, we ascertained if the participants had adopted the clinical behavior targeted in the CPD activity. If so, we asked them to estimate the percentage of relevant clinical cases in which they adopted the clinical behavior. We sent three reminders to all those who agreed to participate in this phase of the study. Participants returned their responses by email to the project manager.

#### Data analysis

Descriptive statistics such as mean, standard deviation, median and frequencies were calculated to summarize participant characteristics and CPD-Reaction questionnaire item responses. Table 1 presents a summary of the score calculations for each item and construct of the CPD-Reaction questionnaire. Briefly, each item response format was pre-coded with Likert-type scale values (Table 1). The item score for each participant ranges from 1 to 7. A score for each construct was obtained by calculating the mean score for the construct (e.g., if the construct includes 2 items, the item scores were summed and divided by 2, yielding a score between 1 and 7).

**Table 1:** Summary of CPD-Reaction questionnaire scores on items and constructs.

Construct scale	Items^a^		Responses choices	Pre-coded item value^b^	Final item score^c^	Score by construct^d^
Intention	I_1_	I intend to [*behavior*]	Strongly disagree/agree	1 to 7	1 to 7	(I_1_+I_7_)/2
	I_7_	I plan to [*behavior*]	Strongly disagree/agree	1 to 7	1 to 7	
Social influence	I_2_	To the best of my knowledge, the percentage of my colleagues who [*behavior*] is…	0–20%	1	1.4	(I_2_+I_6_+I_9_)/3
			21–40%	2	2.8	
			41–60%	3	4.2	
			61–80%	4	5.6	
			81–100%	5	7	
	I_6_	Now think about a co-worker whom you respect as a professional. In your opinion, does he/she [*behavior*]?	Never/Always	1 to 7	1 to 7	
	I_9_	Most people who are important to me in my profession [*behavior*]	Strongly disagree/agree	1 to 7	1 to 7	
Beliefs about capabilities	I_3_	I am confident that I could [*behavior*] if I wanted to.	Strongly disagree/agree	1 to 7	1 to 7	(I_3_+I_5_+I_11_)/3
	I_5_	For me, [*behavior*] would be…	Extremely difficult/easy	1 to 7	1 to 7	
	I_11_	I have the ability to [*behavior*]	Strongly disagree/agree	1 to 7	1 to 7	
Moral norm	I_4_	[*Behavior*] is the ethical thing to do.	Strongly disagree/agree	1 to 7	1 to 7	(I_4_+I_10_)/2
	I_10_	It is acceptable to [*behavior*]	Strongly disagree/agree	1 to 7	1 to 7	
Beliefs about consequences	I_8_	Overall, I think that for me [*behavior*] would be…	Useless/Useful	1 to 7	1 to 7	(I_8_+I_12_)/2
	I_12_	Overall, I think that for me [*behavior*] would be…	Harmful/Beneficial	1 to 7	1 to 7	

^a^ Item number (e.g., I_1_ = Item 1)

^b^ Pre-coded item value is a Likert scale assigned value (i.e., Strongly disagree = 1, Strongly agree = 7; Never = 1, Always = 7, etc.)

^c^ Final item score is the score by item for each participant (possible range scale = 1 to 7)

^d^ Score by construct = mean score by construct (possible range scale = 1 to 7).

Note: for constructs with two items, no imputed values are possible. For constructs with three items, the raw score of the scale is missing if two or more items are missing. In the case of one missing item, the missing item is imputed from the mean of the two other item.

The responsiveness of the CPD-Reaction questionnaire to change in behavioral intention was examined by comparing the mean ranks of each construct obtained before and after CPD activities using the Wilcoxon signed-rank test. Data collected three months after the CPD activities were used to estimate how effective the tool is for predicting behavior. Based on the results of a systematic review of factors predicting behavior in health professionals [11], self-reported behavior can be used as a proxy of behavior change. Predictive validity was estimated by comparing the intention (median *intention* construct score) of health professionals who reported a behavior change three months after the CPD activities with the intention score of those physicians who reported no change, using a Mann-Whitney test. We considered a p value < .05 as statistically significant. Analysis was performed using SAS version 9.3 software (SAS Institute Inc., Cary, North Carolina).

### Qualitative study

#### Participants and recruitment strategy

To assess the barriers and facilitators for implementing the CPD-Reaction questionnaire in CPD settings, we conducted semi-structured phone interviews with CPD providers involved in the development of these activities in the province of Quebec, Canada. Members of the research team identified key opinion leaders (or key individuals to contact). An invitation was sent by email to all potential participants.

#### Data collection

The interview guide was pre-tested with a member of the research team (ML) who has many years of experience as a CPD trainer. Those who had agreed to participate received the semi-structured interview guide by email, a sample of the generic CPD-Reaction questionnaire, and an example adapted to the context of a specific CPD activity. Participants were instructed to review these documents before the interview. Phone interviews were conducted individually in English or French. Before the beginning of each interview, a brief summary of the research project was presented to each participant to contextualize the CPD-Reaction questionnaire and explain the purpose of the interview. All interviews were audio recorded and transcribed in full. We conducted the interviews until data saturation was reached [21].

#### Data analysis

We used NVIVO 9 software (QRS International, Melbourne, Australia) to analyse the interview data. Two independent and experienced RAs trained in qualitative analysis identified the themes and sub-themes emerging from the data using a deductive approach. After independent analysis of six interviews, a common coding structure was established through discussion and then applied to the remaining ones. We performed thematic analysis of the content by reducing data, displaying data, and then drawing conclusions [22]. Specifically, we coded the transcripts using three broad categories concerning the instrument: 1) characteristics; 2) usability; and 3) acceptability. The strength of a particular viewpoint was determined by tabulating the number of individuals who expressed it during the interviews. Participants also completed a questionnaire providing sociodemographic data, the characteristics of their CPD organizations, and their function within these organizations.

## Results

### Quantitative study

#### Participants and CPD activities characteristics of the before-and-after study

**Fig 1:**
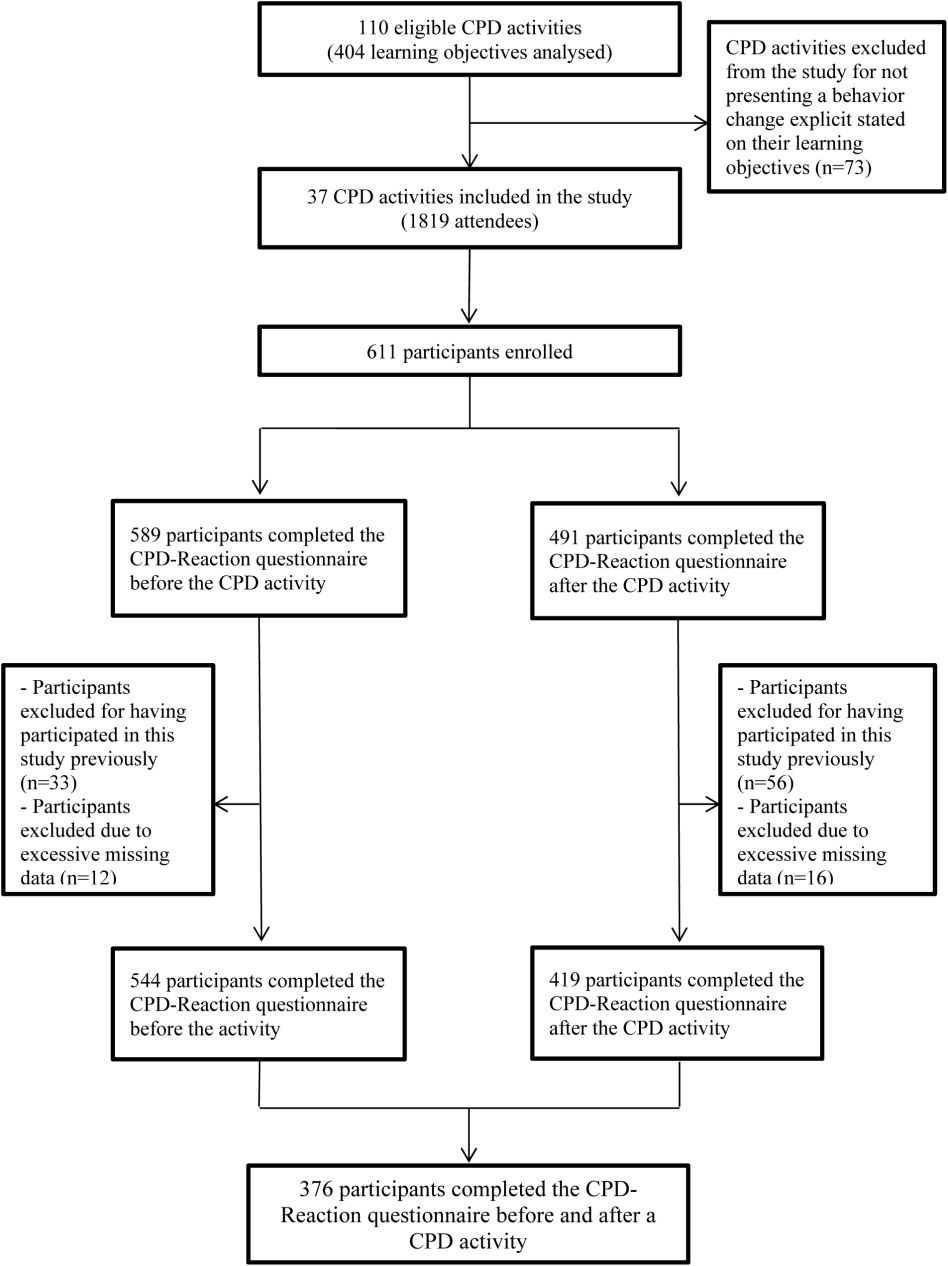
Recruitment flow chart.

Out of 110 eligible activities evaluated, representing a total of 404 learning objectives from the previous study [19], we found 37 activities with at least one learning objective that focused on behavior change for any topic or content. A total of 1819 attendees registered for these 37 activities, of whom 611 were enrolled to participate in this study by a RA just before the activity begun. Most activities were lectures lasting an average of 45 minutes (n = 24), offered within the context of a symposium offering several activities in one setting. Other participants were attending interactive workshops lasting at least three hours (n = 13). Half of the eligible CPD activities were conducted in groups of fewer than 50 attendees (n = 19; range: 11–50) while the other half were conducted in groups of more than 50 attendees (n = 18; range: 61–130). Overall, 376 participants (62%) completed the questionnaire before and after the CPD activity (Fig 1). Half of the participants who completed the CPD-Reaction questionnaire were female. The majority of respondents were family physicians (62%), while 8% were specialists, 5% were residents, and 8% were health professionals other than physicians (Table 2). The characteristics of the CPD activities are described elsewhere [19].

**Table 2:** Characteristics of participants in the before-and-after study.

Characteristics*	Category	N (%) Total = 376 (100%)
Age (years)	20–30	62 (16.5)
	31–40	61 (16.2)
	41–50	83 (22.1)
	51–60	82 (21.8)
	61 and over	25 (6.6)
	NA	63 (16.8)
Gender	Female	186 (49.5)
	Male	125 (33.2)
	NA	65 (17.3)
Professional status	Family physician	234 (62.2)
	Specialty physician	31 (8.2)
	Resident	18 (4.8)
	Other health professionals	30 (8)
	NA	63 (16.8)
Years of clinical practice	N = 261	18.7±11.8

* 17% of participants did not respond to the sociodemographic questions.

#### Responsiveness to change and predictive validity of the CPD-Reaction questionnaire

Table 3 summarizes the descriptive statistics of the items and constructs of the CPD-Reaction questionnaire. Mean and median values were very high before the CPD activities for each construct. The score distributions were non-normal because skewed to the right. Overall, we observed an increase in score for all constructs post-activity (*P* < 0.001) with the most appreciable increase for the construct *beliefs about capabilities* (the median increase of 5 to 6).

**Table 3:** Statistical analysis of each item and construct of the DPC-Reaction questionnaire, scored on a scale of 1–7.

Construct	Item	Before				After				Wilcoxon signed-rank test *P* value
		N	Item mean (SD)	Construct mean (SD)^a^	Median	N	Item mean (SD)	Construct mean (SD)^a^	Median	
**Intention**	I1	374	5.8 (1.3)	5.7 (1.2)	6	376	6.2 (1.1)	6.1 (1.1)	6.5	<0.001
	I2		5.6 (1.3)				6.0 (1.1)			
**Social influence**	SI1	376	4.0 (1.7)	4.8 (1.2)	5	376	4.3 (1.6)	5.0 (1.2)	5	<0.001
	SI2		5.2 (1.3)				5.4 (1.1)			
	SI3		5.2 (1.3)				5.4 (1.2)			
**Beliefs about capabilities**	BCa1	376	5.3 (1.4)	4.9 (1.2)	5	376	6.0 (1.1)	5.8 (1.0)	6	<0.001
	BCa2		4.6 (1.2)				5.4 (1.1)			
	BCa3		4.9 (1.4)				5.9 (1.1)			
**Moral norm**	MN1	375	6.2 (1.1)	6.2 (0.9)	6.5	376	6.4 (0.8)	6.4 (0.8)	7	<0.001
	MN2		6.1 (1.1)				6.4 (0.9)			
**Beliefs about consequence**	BCol1	373	6.0 (1.2)	6.0 (1.0)	6	374	6.2 (1.0)	6.2 (1.0)	6.5	<0.001
	Bcol2		6.0 (1.1)				6.2 (1.0)			

N = number of participants (N varies due to missing data); SD = Standard Deviation; CI = Confidence Interval

^a^ Construct means were calculated as the average of their item scores (possible range scale 1 to 7).

Of the 376 participants in the before-and-after study, 313 participants agreed to be contacted three months later to self-report their clinical behavior. We contacted all 313 participants but only 69 (22% participation rate at follow-up) reported back, of whom 43 (62%) self-reported a clinical behavior change as targeted by their CPD activity. Table 4 (predictive validity) shows the results of comparing the post-activity intention scores of the 43 physicians who reported a change three months later with the scores of the 26 physicians who did not. We did not observe a statistically significant difference between these two groups (*P* = 0.30). However, we observed that in both the pre and the post assessment period, the group who self-reported that they had changed their behavior had higher scores than the group that did not. Also, the group who reported a behavior change three months later presented a potential ceiling effect concerning their intention to adopt the behavior proposed by the CPD activity.

**Table 4:** Prediction of change in behavioral intention scores three months after the CPD activities.

		Before			After				
Construct	Self-reported behavior change	N	Mean (SD)	Median	N	Mean (SD)	Median	Wilcoxon signed rank test^a^	Mann_Whitney test *P* value^b^
Intention	Yes	43	6.03(1.08)	6.50	43	6.34 (0.90)	7.00	0.002	0.30
	No	26	5.33 (1.58)	5.50	26	6.04 (1.04)	6.50	0.011	

^a^ Comparison of behavioral intention mean ranks of physicians obtained before and after CPD activities (paired data).

^b^ Comparison of behavioral intention median between physicians who reported behavior change three months after CPD activities and those who reported no change (independent data).

### Qualitative study

#### Participants’ characteristics

Of the 31 CPD decision-makers and providers who were emailed invitations, 16 replied and accepted the invitation. Data saturation was reached with eight interviews. Then we conducted three more interviews (n = 11). At this point, as we did not observe any new ideas emerging from the interviews, we ceased contacting participants. The majority of participants were physicians (n = 10), male (n = 10), aged 51 years and older, and had been involved in CPD activities, on average, for 23 years. Most participants were representatives of CPD organizations (n = 7) from either a medical regulatory body (n = 2), or academic institutions (n = 5) in the province of Quebec, Canada. Three participants were keynote speakers invited by CPD organizations to develop activities concerning their medical speciality. One participant worked directly with CPD evaluations in a medical school.

#### Acceptability of the CPD-Reaction questionnaire among CPD decision-makers and providers

Most respondents (n = 7) found the CPD-Reaction questionnaire relevant for the evaluation needs of their organizations. It was considered to be a helpful tool for understanding how a specific clinical behavior is adopted as a result of a CPD activity. Four respondents criticized the repetitive nature, or redundancy, of the CPD-Reaction questionnaire items.

While most respondents (n = 8) found the CPD-Reaction questionnaire to be adaptable to the CPD activities offered by their organizations, the usability of the instrument in different CPD contexts polarized respondents’ opinions. Half of respondents (n = 5) were convinced that the instrument could be used in all CPD contexts. Some participants (n = 3) even suggested other uses than the one initially proposed, (i.e., the assessment of the impact of a CPD activity on clinical practice). Some mentioned that their CPD organizations could use the instrument to validate the learning objectives of a new CPD activity and when needed, modify the objectives in order to indicate the behavior to adopt after the activity. However, the other half (n = 6) found that the usefulness of the CPD-Reaction questionnaire would be restricted only to CPD activities focusing on behavior change.

Although most people involved with the planning and organization of CPD activities had a positive attitude towards the use of the CPD-Reaction questionnaire, respondents were unanimous in affirming that participants attending CPD activities would not be favorable to regular use of the instrument. The main reason for this would be physicians’ habitual resistance to completing evaluation forms and the time consumed to complete it (Table 5).

**Table 5:** Barriers and strategies related to the implementation of the CPD-Reaction questionnaire to evaluate the impact of CPD activities on clinical practice.

Barriers identified	Strategies
Lack of interest among all participants in completing evaluation forms after a CPD activity	Encourage participants to complete the instrument by making it part of the activity or a requirement for receiving the credits associated with the activity.
Lack of time during most CPD activities to complete the instrument	Convince CPD planners to allot enough time for participants to complete the instrument, as part of the activity.
Repetitive nature of the theory-based instrument	Simplify the instrument by decreasing its number of items per construct. A shorter instrument would encourage participants attending a CPD activity to complete it.
Adapting the theory-based instrument to each activity	Create a manual containing all necessary information for adapting the instrument to different CPD activities, and how to interpret the results.

As for strategies that could be used for overcoming identified barriers, respondents said it was important to convince participants to complete the instrument. Some of them (n = 4) suggested that the completion of the CPD-Reaction questionnaire should be mandatory to receive credit for attending the CPD activity. Some (n = 4) suggested that the research team advertise the CPD-Reaction questionnaire to encourage its widespread use among representatives of CPD organizations.

## Discussion

This study investigated the responsiveness of the CPD-Reaction questionnaire to behavioral intention change after completing a CPD activity and its predictive validity for actual behavior change in clinical practice. It also verified its acceptability among CPD stakeholders to help the research team to plan for an implementation strategy. Overall, our results indicate that the CPD-Reaction questionnaire is sensitive to changes in behavioral intention and its determinants before and after CPD activities. We observed no statistically significant results pertaining to its predictive validity for actual behavior change, but observed that the median intention scores tended to be higher among health professionals who reported they had adopted the behavior proposed by the CPD activity than among those who had not adopted it. In addition, diverse CPD stakeholders said that CPD-Reaction was of interest to them but that a future implementation strategy would need to address identified barriers, mostly time to complete it. These results lead us to make four main observations.

First, our analysis indicated that the CPD-Reaction questionnaire is responsive enough to detect a change in the behavioral intention of health professionals attending CPD activities. In general, results confirmed that health professionals tend to stay within their “comfort zones” when selecting their CPD activities, aiming most of the time to confirm or verify that their practice is the same (or similar enough) to what their peers are doing [23,24]. However, this does not necessarily mean that CPD activity persuades health professionals to actually change how they practice medicine. Indeed, most CPD activities do not target clinical behavior change and/or patient outcomes [19].

Second, the integrated conceptual model upon which the CPD-Reaction is based proposes that behavioral intention is a predictor of behavior. Interestingly, both pre- and post-activity median intention scores tended to be higher among health professionals who reported that they had adopted the behavior proposed by the CPD activity than among those who had not adopted it, which suggests a confirmation of the integrated conceptual model. However, we did not find statistically significant results pertaining to predictive validity of the CPD-Reaction questionnaire between the groups. This lack of statistical significance could be explained by a considerable ceiling effect in the behavioral intention construct scores and a very low response rate at follow-up for which we obtained an effect size (the rank-biserial correlation) of 0.18, a negligible effect size for adequate power [25]. Future research should take into account reproducibility of the methods but also consider proper rewards and compensations for participants in the longitudinal portion of the study to maximise sample size and increase statistical power.

Third, we found that the CPD-Reaction questionnaire was accepted well by CPD stakeholders interviewed, who believed that the CPD-Reaction questionnaire was relevant for the evaluation needs of their organizations. This relevance may be due to trends in the CPD field, mainly new accreditation regulations (such as the Mainpro+ quality criteria requirements of the College of Family Physicians of Canada and forthcoming National Accreditation standards) that emphasize evaluation and outcome assessment and knowledge transfer tools. CPD stakeholders did, however identify various barriers to its implementation in CPD settings. These barriers were mostly participants’ limited interest in completing evaluation forms after a CPD activity, which could have been aggravated by the apparent redundancy of some of the CPD-Reaction items: few CPD participants may be familiar with social cognitive theories and the measurement needs of this type of instrument. These barriers are not surprising as they are observed in other types of innovative implementation in clinical practices [26,27]. Future research is necessary to develop efficient strategies for overcoming these barriers and for increasing participants’ motivation to complete the questionnaire.

Fourth, the CPD-Reaction questionnaire has provoked a new interest in assessing CPD activities. Such assessments may result in a new form of CPD design among CPD stakeholders. Producing effective CPD activities will require identifying relevant determinants of behavior change and formulating learning objectives that focus on the higher cognitive skills [28] in order to promote a change in professional practice and in patient outcomes. This could lead to the development of more efficient CPD activities that focus more directly on results in changes in practice and improvements in patient care. Moreover, it would also allow for the target audience to display different levels of readiness to change their practice [29]. A user manual containing all necessary information for adapting the instrument to different CPD activities, and how to interpret the results, is available from the authors.

This study has some limitations. First, because we did not have a control group we could not be sure that the behavior change was due to the CPD activity, or due to a measurement effect (i.e., the act of completing the questionnaire) [30]. Second, the low three-month follow-up of 22% of the initial group of participating doctors is a significant limitation of our study. Third, the small sample size used for the qualitative study may have been insufficient; however, according to the literature, data saturation justifies confidence in the results [21]. Finally, we could not standardize the CPD-Reaction questionnaire scores because the scores were not normally distributed.

## Conclusion

This study represents the last phase of a research program to support the validity of the CPD-Reaction questionnaire for assessing the impact of CPD activities on health professionals’ clinical behavioral intentions. The CPD-Reaction questionnaire is a brief and valid tool that it is able to detect a change in the behavioral intention of health professionals attending CPD activities. The instrument seems to be appropriate for assessing the impact of CPD activities on behavioral intention. CPD providers found it to be relevant for evaluating the activities that they offer to health professionals, although future implementation will require addressing the barriers they identified.

## Supporting information

S1 Table The generic CPD-Reaction questionnaire. (DOCX)
