# Supplementary material for: Responsiveness of a simple tool for assessing change in behavioral intention after continuing professional development activities
Source: PLoS One. 2017 May 1;12(5):e0176678. doi: 10.1371/journal.pone.0176678 (PMC5411052; doi:10.1371/journal.pone.0176678)
Supplement: S1 Table — (DOCX) [file pone.0176678.s001.docx]

S1 Table. The generic CPD-Reaction Questionnaire.

| **Item** | **Response choice** |
| --- | --- |
| 1. I intend to *handle an automated external defibrillator.* | Strongly disagree/agree |
| 2. To the best of my knowledge, the percentage of my colleagues who *handles an automated external defibrillator* is: | 0-100% |
| 3. I am confident that I could *handle an automated external defibrillator* if I wanted to. | Strongly disagree/agree |
| 4. *Handling an automated external defibrillator* is the ethical thing to do. | Strongly disagree/agree |
| 5. For me, *handling an automated external defibrillator* would be: | Extremely difficult/easy |
| 6. Now think about a co-worker whom you respect as a professional. In your opinion, does he/she *handle an automated external defibrillator*? | Never/always |
| 7. I plan to *handle an automated external defibrillator.* | Strongly disagree/agree |
| 8. Overall, I think that for me *handling an automated external defibrillator* would be: | Useless/useful |
| 9. Most people who are important to me in my profession *handle an automated external defibrillator.* | Strongly disagree/agree |
| 10. It is acceptable to *handle an automated external defibrillator.* | Strongly disagree/agree |
| 11. I have the ability to *handle an automated external defibrillator.* | Strongly disagree/agree |
| 12. Overall, I think that for me *handling an automated external defibrillator* would be: | Harmful/beneficial |
